# Intermittent chemotherapy in metastatic androgen-independent prostate cancer

**DOI:** 10.1038/sj.bjc.6601232

**Published:** 2003-09-09

**Authors:** T M Beer, M Garzotto, W D Henner, K M Eilers, E M Wersinger

**Affiliations:** 1 Division of Hematology and Medical Oncology, Department of Medicine, Oregon Health & Science University, Mail Code L586, 3181 SW Sam Jackson Park Road, Portland, OR 97239, USA; 2 Oregon Health & Science University and Portland VA Medical Center, Division of Urology, 3710 SW US Veterans Hospital Road, Mail Code P3GU, Portland, OR 97207, USA

**Keywords:** docetaxel, calcitriol, vitamin D, prostate cancer, chemotherapy

## Abstract

Intermittent use of chemotherapy for androgen-independent prostate cancer (AIPC) instead of treatment until disease progression may reduce toxicity. We prospectively tested this approach in eight AIPC patients responding to calcitriol plus docetaxel who reached a serum prostate-specific antigen (PSA) <4 ng ml^−1^. Chemotherapy was suspended until a rise in PSA⩾50% and 1 ng ml^−1^. The median duration of treatment holiday was 20 weeks (13–43+weeks) and all patients retained sensitivity to re-treatment. Chemotherapy holiday was associated with an improvement of fatigue (*P*=0.05). Intermittent chemotherapy for AIPC is feasible and deserves further study.

Treatment for androgen-independent prostate cancer (AIPC) is rapidly evolving. Chemotherapy with mitoxantrone and prednisone offers a palliative benefit but no survival advantage (Tannock *et al*, 1996; Kantoff *et al*, 1999). Long-term therapy with this regimen is not feasible due to cumulative dose-related cardio-toxicity.

New regimens that have substantial activity in phase II studies and are not associated with cumulative dose-related toxicities have recently been developed. These include single-agent docetaxel; combinations of estramustine with docetaxel, paclitaxel, etoposide, and vinca alkaloids; multiagent chemotherapy; and combinations of biologic agents with chemotherapy (Gilligan and Kantoff, 2002).

The optimal duration of chemotherapy for AIPC, particularly in patients who have had a substantial response to treatment, remains undefined. Most protocols call for treatment until progression. This approach exposes some responding patients to uninterrupted chemotherapy for prolonged periods of time. For example, the median time to progression with docetaxel plus estramustine treatment was 8 months for all patients and 10 months for patients with measurable disease (Savarese *et al*, 2001).

Intermittent hormonal therapy is an approach that may limit the chronic toxicity of this systemic treatment in prostate cancer (Goldenberg *et al*, 1995; Higano *et al*, 1996) and is currently being evaluated in randomised trials. Similarly, intermittent administration of chemotherapy for the treatment of AIPC may reduce treatment-associated toxicity, but no data regarding this approach are available.

In the context of a phase II clinical trial of high-dose calcitriol and docetaxel in AIPC, we sought to develop and evaluate prospectively an intermittent chemotherapy protocol. The rationale for calcitriol plus docetaxel has been previously described (Beer *et al*, 2001). This report is focused on a detailed examination of intermittent chemotherapy.

## MATERIALS AND METHODS

Detailed eligibility criteria and treatment regimen have been previously reported (Beer *et al*, 2001). Briefly, the study was IRB approved and informed consent was obtained from all participants. Men with chemotherapy-naïve, metastatic AIPC were treated with calcitriol (Rocaltrol® 0.5 *μ*g capsules, Roche Pharmaceuticals, Nutley, NJ, USA) 0.5 *μ*g/kg on day 1 followed by docetaxel (Taxotere®, Aventis Pharmaceuticals, Bridgewater, NJ, USA) 36 mg m^−2^ intravenously over 15–30 min on day 2 of each treatment week. Dexamethasone 8 mg orally was given 12 and 1 h prior to and 12 h after docetaxel. Treatment was administered weekly for 6 consecutive weeks on an 8-week cycle.

To be eligible for intermittent chemotherapy, patients had to meet criteria for prostate-specific antigen (PSA) response (50% reduction confirmed 4 weeks apart) and had to have reached a serum PSA<4 ng ml^−1^. During the chemotherapy holiday, disease status was monitored with a serum PSA and clinical examination every 4 weeks and, in patients with measurable disease, by imaging every 8 weeks. Quality of life (QOL) was assessed at the beginning and at the end of the chemotherapy holiday by the EORTC core questionnaire QLQ-C30 (Aaronson *et al*, 1993; Osoba *et al*, 1994) obtained with permission from the EORTC (Brussels, Belgium). Chemotherapy was resumed after a confirmed PSA increase of 50% and of at least 1 ng ml^−1^ or for any other evidence of disease progression.

## RESULTS

In total, 37 eligible patients were registered between May 2000 and May 2001. In all, 30 patients met criteria for PSA response (Bubley *et al*, 1999). Of these, 11 reached confirmed serum PSA < 4 ng ml^−1^. A comparison of pretreatment characteristics between patients who became eligible for intermittent chemotherapy and those who did not is shown in Table 1

**Table 1:** Patient characteristics on entry

**Characteristics**	**Eligible for intermittent therapy**	**Ineligible for intermittent therapy**	**Total**	***P*-value^a^**
No. of patients	11	26	37	N/A
Age (years)				
Median (range)	73 (46–82)	73 (62–83)	73 (46–83)	0.960

*ECOG performance status*				
0	6	7	13	0.347
1	4	11	15	
2	1	7	8	
3	0	1	1	

PSA (ng ml^−1^)				
Median (range)	12 (6–553)	144 (20–921)	99 (6–921)	0.002

*Site of metastases*				
Bone only	8 (73%)	14 (54%)	22 (59%)	0.481
Bone and lymph nodes	2 (9%)	10 (38%)	12 (32%)	
Lymph nodes only	1 (18%)	2 (8%)	3 (8%)	

*Prior radiation*				
External beam to primary	5 (45%)	12 (46%)	17 (46%)	0.969
External beam to metastases	1 (9%)	7 (27%)	8 (22%)	0.229
Radiopharmaceuticals	0 (0%)	1 (4%)	1 (3%)	0.510

No. of prior hormonal treatments				
Median (range)	2 (1–3)	2 (1–3)	2 (1–3)	0.618

a Mann–Whitney *U*-test was used for comparison of continuous variable, *χ*^2^-test was used for comparison of categorical variables.

PSA=prostate-specific antigen.

. The two groups differed only by their baseline PSA concentrations.

Nine patients chose intermittent chemotherapy after receiving a median of 45 weeks of chemotherapy (range 25–53 weeks). One patient was excluded from the study for reasons unrelated to prostate cancer or its treatment. Eight patients were therefore evaluable for outcome with intermittent chemotherapy. The median duration of treatment holiday was 20 weeks (range 13–43+weeks). Seven patients resumed therapy, while one patient remained on break after 43 weeks. In all patients, resumption of treatment was triggered by a rising PSA. After resumption of therapy, four patients met criteria for PSA response from their postholiday PSA and three had stabilisation of the PSA for at least 12 weeks to date. With a median follow-up of 10.1 months from the start of the chemotherapy holiday (and 20.4 months from study entry), the median time to progression has not been reached. Two patients had measurable disease. Both of these entered the break after a partial response (PR) and retained PR status throughout the treatment holiday.

The score for each QOL domain at the end of the break (or last available in the one patient who remains on break) was compared to the same at the beginning of the treatment holiday with each patient serving as his own control. No difference in any of the function scales of QLQ-C30 was found. Statistically significant improvement in fatigue (*p*=0.05 by paired *t*-test) was seen. A trend (*P⩽*0.1) towards improvement in dyspnoea, anorexia, and diarrhoea and a trend towards a worsening in pain were observed. All domains for which a difference with a *P* value ⩽0.1 are shown in Table 2

**Table 2:** Pretreatment QLQ-C30 scores on a scale of 0–100

	**(Mean±s.d. (*n*=7))**		
**Scale**	**Before treatment holiday**	**After treatment holiday**	***P*-value^a^**
Symptom scales^b^			
Fatigue	40.3±23.0	27.8±18.8	0.05
Pain	18.8±24.3	27.1±33.2	0.10
Dyspnoea	33.3±30.9	12.5±17.3	0.09
Anorexia	16.7±25.2	4.2±11.8	0.08
Diarrhoea	45.8±35.4	29.2±21.4	0.10

a Two-sample, two-tailed *t*-test with a comparison between measurements before and after the treatment holiday.

b Higher values for symptom scales indicate more of the symptom. Within an individual patient, a change of⩾10 points is thought to be important because it is easily perceived by patients (Osoba *et al*, 1998, 1999).

.

## DISCUSSION

While durable complete remission remains an elusive goal in the treatment of AIPC, substantial responses to therapy are frequently reported. In this trial, 30% of patients reached a PSA⩽4 ng ml^−1^. Regimens that omit anthracyclines, such as the one used in the current study, are not associated with cumulative dose-related life-threatening toxicity, but asthenia, oedema, neuropathy, hyperlacrimation, onycholysis are just a few examples of toxicities that typically increase with increasing duration of treatment.

This study demonstrates the feasibility of prospectively planned intermittent chemotherapy in such patients. The duration of chemotherapy holiday was clinically meaningful and no patient progressed after chemotherapy was resumed. The QOL data, although limited by sample size and lack of a control arm, suggest meaningful improvements in common chronic toxicities of chemotherapy with a treatment holiday. At the same time, a trend towards increased pain was observed.

Larger studies are needed to further examine the potential benefits and drawbacks of intermittent chemotherapy compared with continuous chemotherapy. Based on the QOL data collected in this study, one might speculate that in selected patients, the intermittent approach may be associated with a reduction in the chronic toxicity of chemotherapy. A larger sample size would be necessary to determine if the observed reductions in chemotherapy toxicity translate into improvements in overall QOL. The trend towards an increase in pain suggests that intermittent chemotherapy may not be desirable in patients who have significant levels of prostate cancer-related pain and are receiving chemotherapy in order to control pain.

This pilot study focused on feasibility and QOL. The impact of intermittent chemotherapy on measures of cancer control, such as time to progression and survival require further study. Similar to ongoing debates about intermittent hormonal therapy, one might speculate that intermittent application of chemotherapy could hasten, delay, or have no effect on the time to emergence of resistant disease. Randomised studies that compare intermittent to continuous hormonal therapy are underway. A similar study of chemotherapy should be considered.

Intermittent chemotherapy is feasible in AIPC patients. Additional study is needed to determine more definitively the contribution of intermittent chemotherapy to the overall efficacy and toxicity of treatment.
